# A Hyperoxic Lung Injury Model in Premature Rabbits: The Influence of Different Gestational Ages and Oxygen Concentrations

**DOI:** 10.1371/journal.pone.0095844

**Published:** 2014-04-22

**Authors:** Roberta Munhoz Manzano, Renata Suman Mascaretti, Valéria Carrer, Luciana Branco Haddad, Aline Rabelo Fernandes, Ana M. A. Reyes, Celso Moura Rebello

**Affiliations:** 1 Integrated College of Bauru, Bauru, São Paulo, Brazil; 2 Pro Matre Paulista Hospital, São Paulo, São Paulo, Brazil; 3 Albert Einstein Jewish Hospital, São Paulo, São Paulo, Brazil; 4 Saint Isabel Hospital, Aracajú, Sergipe, Brazil; 5 School of Medicine - Clinics Hospital University of Sao Paulo, São Paulo, São Paulo, Brazil; The Ohio State Unversity, United States of America

## Abstract

**Background:**

Many animal models have been developed to study bronchopulmonary dysplasia (BPD). The preterm rabbit is a low-cost, easy-to-handle model, but it has a high mortality rate in response to the high oxygen concentrations used to induce lung injury. The aim of this study was to compare the mortality rates of two models of hyperoxia-induced lung injury in preterm rabbits.

**Methods:**

Pregnant New Zealand white rabbits were subjected to caesarean section on gestational day 28 or 29 (full term  = 31 days). The premature rabbits in the 28-day gestation group were exposed to room air or FiO_2_ ≥95%, and the rabbits in the 29-day gestation group were exposed to room air or FiO_2_  = 80% for 11 days. The mean linear intercept (Lm), internal surface area (ISA), number of alveoli, septal thickness and proportion of elastic and collagen fibers were quantified.

**Results:**

The survival rates in the 29-day groups were improved compared with the 28-day groups. Hyperoxia impaired the normal development of the lung, as demonstrated by an increase in the Lm, the septal thickness and the proportion of elastic fibers. Hyperoxia also decreased the ISA, the number of alveoli and the proportion of collagen fibers in the 28-day oxygen-exposed group compared with the control 28-day group. A reduced number of alveoli was found in the 29-day oxygen exposed animals compared with the control 29-day group.

**Conclusions:**

The 29-day preterm rabbits had a reduced mortality rate compared with the 28-day preterm rabbits and maintained a reduction in the alveoli number, which is comparable to BPD in humans.

## Introduction

Bronchopulmonary dysplasia (BPD) is a multifactorial disorder with a pathogenesis that includes prematurity, the requirement for mechanical ventilation, inadequate antioxidant defenses and activation of the inflammatory response through several mechanisms. The most important mechanism for activating the inflammatory response is oxygen exposure [1], but other mechanisms can result in decreased alveolarization [2]–[7].

Due to this complex pathogenesis, many experimental models have been developed by exposing the immature lung to injuries induced by hyperoxia, mechanical stretch, inflammation and genetic modification [8], [9], [10]. These BPD models, included rats, lambs and baboons were essential tools for obtaining a better understanding of this disease [11]–[17]. Rodents are a well suited to model BPD given that the newborn rodent is born during the saccular stage of lung development. It was shown that the exposure of newborn mice to pure oxygen for one week resulted in pulmonary edema and hemorrhage, followed by a chronic repair phase, characterized by fibroblast proliferation and collagen deposition [18]. Oxygen exposure in rodents not only induces lung injury but also disrupts lung structure, affecting alveolarization and vascularization [19]. Although the premature rat model subjected to hyperoxia is advantageous due to its ease of manipulation and lower cost compared with larger-animal models [20], their main disadvantage is the small body size, which is a restriction for the study of lung mechanics and the alveolar and total lung surfactant pool in premature newborns [11]. Furthermore the oxygen concentrations used in most studies exceed the levels of supplementation currently applied to the preterm infant and lack the oxygen fluctuations that are clinically observed in preterm infants, which could potentially induce differences in molecular signaling not reproduced in the experimental setting [21].

Experimental larger-animal models including the preterm lamb and baboons allowed the use of mechanical ventilation for prolonged time, resulting in the creation of novel animal models of BPD [14]–[16]. Preterm baboons and lambs mechanically ventilated and exposed to maternal glucocorticoids and surfactant treatment, allowed the development of lung histopathologic findings of evolving neonatal chronic lung disease, similar to what occurs in preterm infants with BPD [4], [7], [22], [23]. These characteristics include widening and reduction of the alveoli number, a decrease of the alveolar surface area, presence of capillary dimorphism, increase in alveolar septal thickness and a modification in density and disposition of the elastic and collagen fibers, with persistent muscularization of pulmonary arterioles, and increased muscularization of bronchioles [9], [24], [25]. The preterm baboon program ended almost a decade ago, due to the high cost and ethical concerns, leaving the preterm lamb model as the only large animal, physiological model of neonatal chronic lung disease [26]. Advantage of both models is that they reproduce the clinical setting of preterm birth, respiratory failure, and prolonged ventilation support with oxygen-rich gas for days or weeks [27], [28]. Advantages of the preterm baboon model were the close phylogeny of baboons to humans and the preterm baboons were more immature than preterm lambs. An advantage of the preterm lamb model is that the fetal lambs are larger (2–3 kg body weight) and therefore, more amenable to physiological studies and repeated blood sampling. Disadvantages of both models are that they are expensive and require 24 h critical care.

The use of rabbits as animal models to study the effects of hyperoxia on the developing lung has been limited to lung or macrophage cultures exposed to oxygen. In vivo studies with rabbits following premature birth has been restricted to short periods of hyperoxia ranging from 20 hours to 4 days [29], [30].

In a previous study, pregnant rabbits were inoculated with *Escherichia coli* on the 29^th^ day of gestation, and ceftriaxone therapy was initiated 8 hours after inoculation and continued for a period of 8 days. Although these animals remained under observation for a prolonged period (8 days), the prenatal infection was used to induce lung injury. These studies [10] support the central hypothesis of the current study of using premature rabbits born at 29 days of gestation for an experimental model of BPD.

Premature rabbits born at 28 days of gestation have been used in a model of BPD that was initially developed at our institution. This model was based on long periods of exposure to high oxygen concentrations (FiO_2_ ≥95%). However, the mortality rate was high in these studies [6], [31].

The aim of the present investigation was to evaluate the effects of prolonged exposure to oxygen on the mortality and lung development of premature rabbits by comparing 2 models of lung injury based on different gestational ages and oxygen concentrations: 28-days gestation preterm rabbits exposed to FiO_2_ ≥95% and 29-days gestation preterm rabbits exposed to FiO_2_  = 80%.

## Methods

The experimental protocol was approved by the Ethics Committee for the Analysis of Research Projects at the Teaching Hospital of the University of São Paulo's School of Medicine (CAPPesq - project number 487/07).

On the 28^th^ or 29^th^ day of gestation (full term  = 31 days), pregnant New Zealand white rabbits were subjected to cesarean sections following sedation with ketamine and acepromazine (10 mg/kg and 0.1 mg/kg, respectively, given intramuscularly (IM) and a spinal block using 2% Marcaine and xylocaine (1∶1, vol:vol)).

The abdominal and uterine walls were sectioned, and the fetuses were removed according to their location in the uterine horns (proximal or distal) and identified by the order of their removal. Sequential numbering by marker pen on the dorsal region was used to identify the animals to minimize the effect of the uterine position on the birth weight [32].

The first animal from each litter was randomly assigned to a study group immediately after birth. The groups were composed of animals exposed to room air or animals exposed to oxygen concentrations equal to 80% or ≥95%. Based on the allocation of the first animal from each litter, the animals were alternately allocated to the study groups. Four study groups were created: 28 days of gestation exposed to room air (Air 28), 28 days of gestation exposed to FiO_2_ ≥95% (O_2_ 28), 29 days of gestation exposed to room air (Air 29) and 29 days of gestation exposed to FiO_2_  = 80% (O_2_ 29).

The animals were maintained in premature infant incubators (C-86, Fanem LTDA, São Paulo, Brazil) on trays coated with a thin layer of autoclaved sawdust (sterile wood shavings) that was changed twice per day to keep the animals in a clean, dry environment. The incubator temperature was maintained between 30 and 32°C and was controlled by an electronic thermometer. The animals were weighed daily prior to the first feeding of the day (analytical scale TR 403; Denver Instrument Company, USA).

The animals were fed with a milk formula similar to natural rabbit milk, consisting of 5 g Neocate (Support), 5 g caseical (Support) and 15 g triglyceryl CM AGE (Support) in 100 ml milk. One drop of Vitanove vitamin complex was added to the formula starting on the third day of life.

The diet volume was 100 ml/kg on the first day of life, 150 ml/kg on the second day of life and 200 ml/kg from the third to the eleventh day. Food was administered at 12-hour intervals.

Warm humidified oxygen was supplied at a concentration of 80% or ≥95% to the animals in the O_2_ 28 and O_2_ 29 groups, respectively. The oxygen was delivered by neonatal nebulizers (Intermed, São Paulo, Brazil) through a multiperforated sealed acrylic chamber to prevent the accumulation of carbon dioxide (CO_2_). The oxygen concentrations were controlled using a room oxygen analyzer (Dixtal, São Paulo, Brazil).

The animals were sacrificed by exsanguination through sectioning of the abdominal aorta after achieving deep sedation with pentobarbital (25 mg/kg given intraperitoneally) on the 11^th^ day after birth.

For the histological analysis of the lungs, a tracheostomy was performed with the insertion of a metal cannula (1 mm in diameter) immediately after sacrifice. The lungs were inflated to a pressure of 30 cmH_2_O [33]; after one minute, the trachea was ligated using a 0-0-cotton thread, followed by dissection and removal of the lungs and assessment of the lung volume. The slide preparation involved 1 mm thick sagittal cuts of the distal portion of the right inferior lobe. The author responsible for the morphometric analyses was blinded to the groups.

The histology slides were stained with hematoxylin and eosin for morphometric analysis, modified Weigert's resorcin and orcein for the analysis of elastic fibers and Picrosirius for the analysis of collagen fibers.

All analyses were performed by the same author, at the same time. A Nikon E600 microscope coupled to a 100-point grid of 50 lines was used for the morphometric analysis, and a minimum of 10 microscopic fields were examined per animal at 100× magnification. The morphometric analysis of the slides was performed by 2 blinded investigators.

Lung development was assessed by determining the mean linear intercept (Lm), the internal alveolar surface area (ISA), the number of alveoli, the thickening of the inter-alveolar septum and the proportion of elastic and collagen fibers.

The same 100-point grid of 50 lines was used for the analysis of Lm, considering the intersection of each of the 50 lines in the grid with an alveolar wall as 1 intercept. The Lm was calculated by the following formula: Lm  =  total length of the lines/number of alveolar intercepts.

The ISA represents the alveolar surface that is available for gas exchange and was determined according to the following formula: ISA  = 4 V/Lm, where V =  the fixed lung volume at a trans-tracheal pressure of 30 cmH_2_O multiplied by the parenchymal fraction. The parenchymal fraction was calculated using the same grid by counting the points that overlaid the lung tissue and excluding those that overlaid blood vessels and bronchi larger than 2 mm in diameter. The number of points was divided by 100. The following formula was used: V =  (total lung volume x mean number of points)/100.

The number of alveoli was determined by a blinded investigator using an image analyzer (Image-Pro Plus 4.5, Media Cybernetics INC, USA). Lung sections stained by hematoxylin and eosin were photographed at 100× magnification, and 10 fields (images) were used for each slide.

The thickness of the inter-alveolar septum was determined manually with an image analyzer (Image-Pro Plus 4.5, Media Cybernetics INC, USA) by a blinded investigator. The assessment was performed on one slide per animal that was stained by Picrosirius, and 4 to 8 septa were assessed using 4 photographs per slide at 200× magnification.

The proportion of elastic and collagen fibers in the lung tissue was assessed by a single blinded investigator using an image analyzer (Image-Pro Plus 4.5, Media Cybernetics INC, USA). The lung sections stained by resorcin and orcein (for elastic fibers) and Picrosirius (for collagen fibers) [21] were photographed at 200× magnification, and 20 fields (images) were examined for each slide. The proportion of elastic and collagen fibers was determined by dividing the area of elastic or collagen fibers by the total area minus the area of the airspace.

The survival analyses were performed using the Kaplan-Meier method. Continuous variables were analyzed using one-way ANOVA with the Student-Newman-Keuls test used as a discriminatory post-hoc test. The Kruskal-Wallis one-way analysis of variance by ranks test was used as a nonparametric data test when the data failed to meet the prerequisites for a normal distribution and similar variance. Dunn's method was used as a discriminatory post-hoc test. A level of significance of 0.05 was adopted. SigmaPlot 11.0 (Systat Software, Inc. Chicago, USA) was used for the statistical analysis.

## Results

### Animal Survival

**Figure 1:**
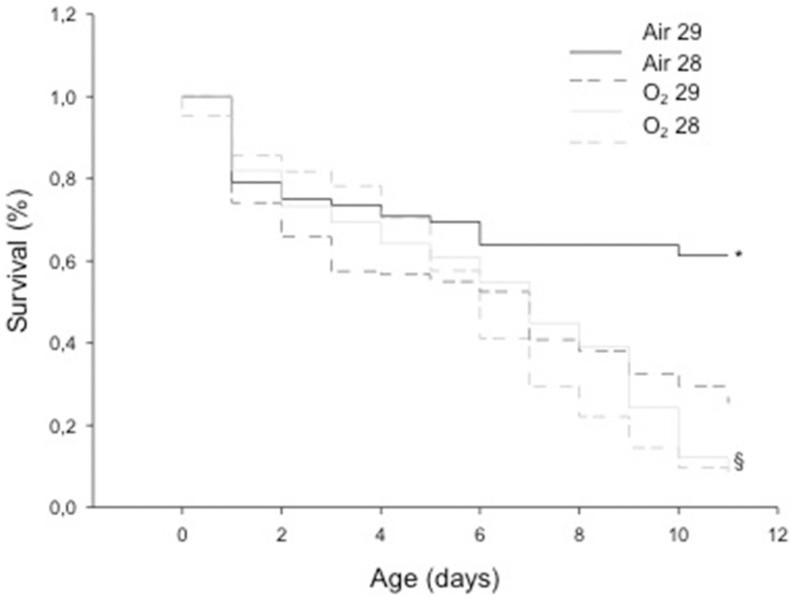
Kaplan-Meier survival curves – A comparison between the groups ‘room air’ and ‘oxygen’ is shown with animals born at 28 (dashed lines) and 29 days of gestation (solid lines). A higher survival rate was observed in both Air Groups (*, p<0.05 vs Oxygen Groups) and a higher survival rate among O2 29 animals vs O2 28 Group (§, p<0.05)

The total number of litters used in this experiment was 65 for the 28-day gestational age pups and 40 for the 29-day gestational age pups, with a mean number of pups/litter of 5.2±2.8 (mean ± sd). The animals in the Air 29 group exhibited higher survival rates compared with the Air 28, O_2_ 28 and O_2_ 29 groups. The survival rate observed in the animals from the O_2_ 29 group was higher than that in the animals from the O_2_ 28 group (Figure 1).

### Morphometric Analysis of the Lungs

The normal development of the lungs was blocked by hyperoxia, as demonstrated by an increased Lm in the O_2_ 28 and O_2_ 29 groups, representing a larger alveolar size, compared with the Air 28 and Air 29 groups. This difference was significant only in the first group. A decrease in the ISA was observed in both groups exposed to oxygen (O_2_ 28 and O_2_ 29) compared with the corresponding groups kept in room air (Air 28 and Air 29), although this difference was significant only in the first group. A significantly decreased number of alveoli and an increased thickness of the inter-alveolar septum were noted in the groups subjected to hyperoxia compared with those kept in room air, thus confirming the interrupted alveolar development secondary to oxygen exposure (Table 1).

**Table 1:** The values of Lm and ISA and the number of alveoli and septal thickening are shown in the groups.

	Air 28	O_2_ 28	Air 29	O_2_ 29
	(n = 17)	(n = 17)	(n = 8)	(n = 8)
**Lm (µm)** 1	60.5±18.2*	85.3±27.3	74.3±10.6	86.4±14.9
**ISA (µm^2^)^2^**	0.033±0.014*	0.031±0.014	0.049±0.020	0.044±0.022
**Alveoli number (per field)^3^**	32.02±11.29^#^	17.91±8.73	30.24±8.46^#^	21.44±4.93
**Septal thickening (µm)^4^**	3.90±1.40^#^	19.08±16.8^§^	3.84±1.09	13.59±6.68
**Lung volume (ml)^5^**	2.63±1.83	1.93±0.80	3.90±1.27	2.81±1.54

*p<0.05 vs. all other groups; ^#^p<0.05 vs. O_2_; ^§^p<0.05 vs. Air 28 and Air 29.

1 : Power of performed test with alpha  = 0,050∶0,975; ^2^: Power of performed test with alpha  = 0,050∶1,000; ^3^: Power of performed test with alpha  = 0,050∶1,000; ^4^: Power of performed test with alpha  = 0,050∶1,000; ^5^: Power of performed test with alpha  = 0,050∶0,087.

### Proportion of Elastic and Collagen Fibers

**Figure 2:**
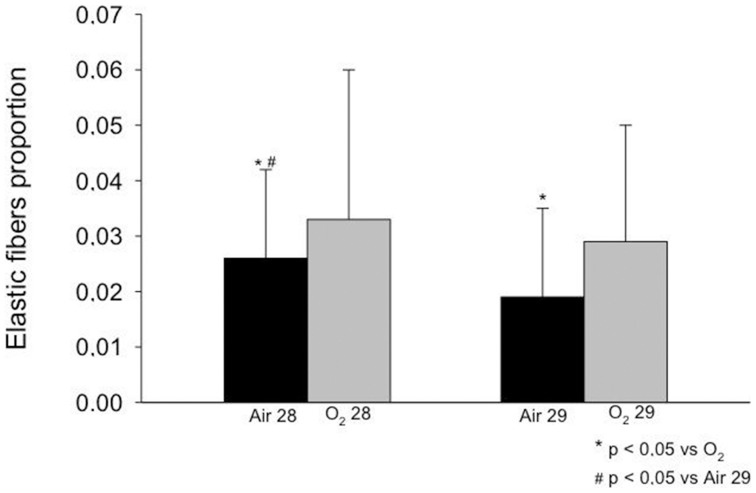
Elastic fibers proportion observed in the study groups. A higher proportion of elastic fibers were observed in both oxygen exposed groups compared with room air at the same gestational age (p<0.05) and a higher proportion of elastic fibers was observed among more immature animals exposed to room air (p<0.05).

**Figure 3:**
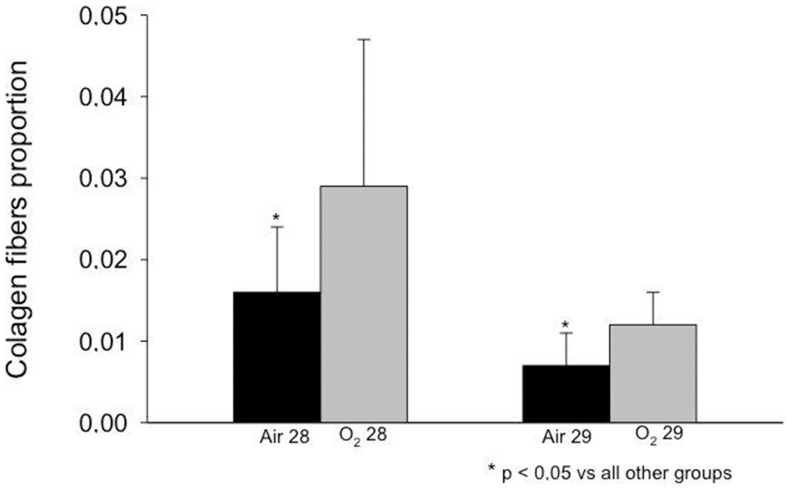
Collagen fibers proportion observed in the study groups. A higher proportion of collagen fibers were observed in both oxygen exposed groups compared with room air at the same gestational age (p<0.05)

**Figure 4:**
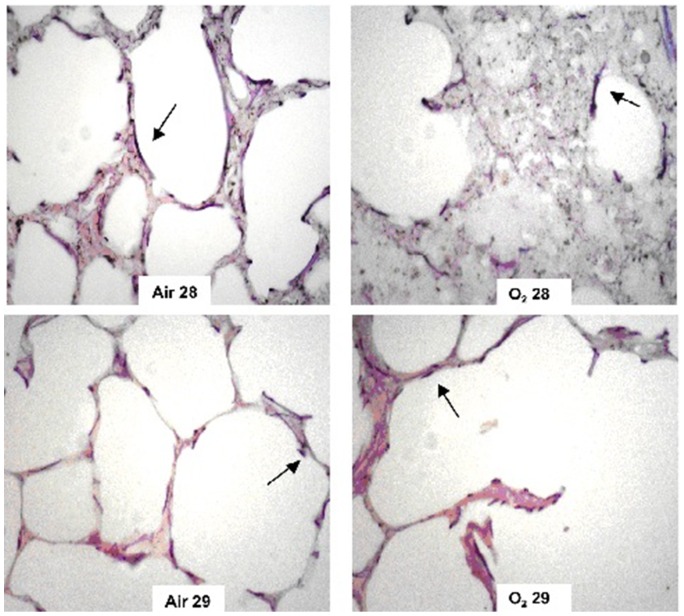
Distribution of elastic fibers (arrows) in the study groups. Oxygen exposed animals (right panels, upper and lower) showed an intense disorganization of elastic fibers compared to animals from the Air 28 and Air 29 group (left panels, upper and lower).

**Figure 5:**
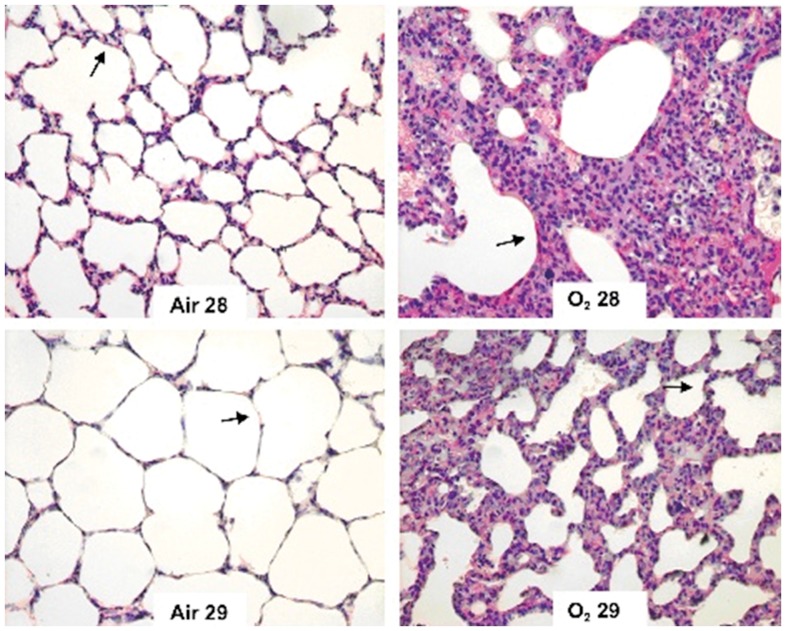
Distribution of collagen fibers (arrows) in the study groups. Animals exposed to oxygen showed an increased proportion of collagen fibers compared to those exposed to room air.

The elastic and collagen fiber proportion increased in the O_2_ 29 group compared with the Air 29 group (Figures 2 and 3). A similar increase in the fiber proportion was observed in the animals in the O_2_ 28 group compared with the Air 28 group (Figures 2 and 3). When both groups of animals born at 28 days were compared with the groups of animals born at 29 days, the elastic and collagen fiber proportion in the 29-day groups was lower than in the 28-day groups. Disorganization of the elastic and collagen fibers was observed in animals that had been exposed to oxygen (Figures 4 and 5, respectively).

## Discussion

The use of premature rabbits born at 29 days of gestation and subjected to an oxygen concentration of 80% resulted in a reduced number of alveoli and a trend in other histopathological findings, comparable with BPD characteristics in humans. These findings were similar to those observed in animals born at 28 days of gestation and subjected to an oxygen concentration ≥95%, though with a higher survival rate. A small difference of one day in the gestational age caused differences (comparable to hyperoxia) in the morphometric analysis of the lungs.

The use of premature rabbits exposed to hyperoxia as an additional experimental model to study BPD has the advantage of a lower cost, when compared with the baboon or preterm lamb model, which require intensive care for 24 hours, and a structure of complex and expensive laboratory not available in many research centers [15], [34]. Furthermore, the size of the premature rabbit (approximately 33±5 g), which is larger than the preterm mouse or rat, allows the use of a number of injuries, including hyperoxia, mechanical ventilation [35], and inflammation [36], that make this model suitable not only to better understand different molecular pathways which result in impaired lung development, with disruption of the entire process of alveolarization, but also the pathways that mediate lung repair and regeneration after injury. Future refinement and modification of this current animal model to better mimic the human condition is important, including to limit the levels of O_2_ used to cause lung injury to those currently used in preterm infants, as well as to allow fluctuations of O_2_ levels [37] and the use of therapies more similar to current standard of care (steroids, surfactant, etc.) [38].

This animal model shows close concordance to human beings in terms of the stages of lung development [30], [32] because the alveolar phase of rabbit development begins at the 28^th^ day of gestation, for a full term of 31–33 days. This alveolar phase continues for a long period after birth, with a dramatic increase during the first 2 weeks of postnatal life; therefore, there may be critical differences between species in the early control of alveolarization and, thus, the pathways leading to lung injury [32], [39]. With 29 days of gestation, most of the alveolarization has not occurred, which makes this timing an acceptable model for BPD in humans, although with a lower mortality rate. This model was previously used to evaluate the impact of postnatal nutrition on lung growth and development [40].

The decision to use oxygen as an aggravating factor was based on the ease of implementing this model and on previous studies demonstrating that oxygen alone can trigger the cascade of inflammatory events that interrupt alveolar development. Rat lungs exposed to 100% oxygen until death or 85% oxygen for 7 days exhibit proliferation and hypertrophy of type II epithelial cells, decreased surface area and capillary volume and increased superoxide dismutase activity [18], [41]. Premature newborns are particularly sensitive to the deleterious effects of oxygen [11]. The ability of preterm infants to manage free radicals found in the plasma is low and declines during the early postnatal period [8], [42].

Our results show that survival was higher among animals born at 29 days of gestation compared with those born at 28 days of gestation among both animals exposed to oxygen and those subjected to room air. The higher survival rate in the former group may hinder the practical use of this experimental model [31]. A 67% mortality rate was observed in rats exposed to a FiO_2_ ≥95% for 14 days compared with rats exposed to room air (the mortality rate at room air was 33%) [11].

To characterize pulmonary lesions caused by hyperoxia, we studied the major changes that occur in BPD in humans, including changes in the alveolar size, the ISA, the number of alveoli, the thickness of the alveolar septum and the proportion of elastic and collagen fibers in the pulmonary parenchyma [13].

The size and distance between the alveolar walls is well represented in a simplified analysis through determining the Lm. In this study, hyperoxia modified the normal development of the lung, as demonstrated by an increase in the Lm. A similar evaluation of the interruption of lung development was performed in premature rats treated with dexamethasone or saline solution 12 hours before birth and randomized to hyperoxia (≥95%) or room air immediately after birth for 7 to 12 days [11]. Another similar evaluation was performed in premature baboons subjected to mechanical ventilation and hyperoxia [15].

A study conducted on 8 children with fatal cases of BPD also found an increased Lm, demonstrating the importance of this parameter in characterizing the disease [23].

In a model previously developed at our institution, premature rabbit lungs exposed to hyperoxia (≥95%) for 7 or 11 days exhibited increases in Lm compared with the room air group [6], [31]. In the present study, the O_2_ 28 group showed histopathological findings that are consistent with the majority of studies in the literature, which report a 24% increase in Lm associated with oxygen exposure. This increase represents an increase in alveolar size secondary to impaired lung development, and the O_2_ 29 group showed a similar trend with a 16% increase [6], [11], [23], [31].

The ISA calculation reflects the area of the alveolar-capillary membrane and the surface area that is available for performing gas exchange [43], [44]. This study demonstrated a decreased ISA in the groups that had been exposed to oxygen (O_2_ 28 and O_2_ 29) compared with the groups subjected to room air, although significance was reached only in the O_2_ 28 group. Several authors have studied the effects of the impaired lung development observed in BPD on the ISA. Rats exposed to hyperoxia (95%) for 7 days exhibit a decreased ISA compared with rats exposed to room air [11]. Baboons treated with exogenous surfactant after birth and exposed to oxygen and mechanical ventilation show a 60% decrease in the ISA [15].

The autopsies of newborn infants with BPD have indicated a reduced ISA [23], confirming the importance of analyzing this parameter in addition to Lm in experimental models of this disease.

The reduced number of alveoli in the lungs consequent to impaired lung development is a central finding in BPD. Rats exposed to FiO_2_ 60% exhibit a 50% reduction in the number of alveoli [45]. Interrupted alveolar development has been demonstrated in premature baboons (at 71% gestation) that developed lung injury following ventilation without volutrauma [43] and in baboons after premature birth and mechanical ventilation with 80–100% FiO2 for 3 weeks [16], [17]. A study on premature lambs subjected to ventilation for 3 or 4 weeks revealed reduced alveolar septation and fewer alveoli compared with animals mechanically ventilated after birth at term [13].

Newborn mice exposed to room air or 85% oxygen for 28 days exhibit decreased alveolar septation, an increased number of terminal air spaces and increased pulmonary fibrosis in oxygen group. These findings are evident after 7 days of oxygen exposure and are more pronounced at 28 days [46].

The autopsies of premature infants with BPD demonstrated a reduced number, area and perimeter of alveoli. In this study, the finding of a reduced number of alveoli in the groups subjected to hyperoxia demonstrates that animals born at 29 days of gestation and exposed to 80% oxygen for 11 days had reduced mortality and reproduced this central characteristic of BPD [47].

Regarding the thickness of the alveolar septum, an increase was noted in the O_2_ 28 and O_2_ 29 groups compared with the Air 28 and Air 29 groups, although similar to the ISA, significance was reached only in the O_2_ 28 group. Increased thickness of the alveolar septum has been described in newborn infants with fatal cases of BPD [23]. Several studies have confirmed the above-described findings in humans regarding the thickness of the alveolar septum and its relationship with BPD [16], [17].

The effect of BPD on the proportion of elastic fibers in the premature lung has been extensively studied. This study found an intense disorganization and increased proportion of elastic fibers in animals from the O_2_ 28 group compared with the Air 28 group. The proportion of elastic fibers was smaller in the 29-day groups (Air and O_2_) than in the 28-day groups. Some authors have described an increased deposition of elastic fibers in the alveolar walls and an increased and abnormal distribution of elastin in the terminal respiratory units among the pathological findings of BPD in children [15], [48], [49]. Other authors have reported that the elastic tissue in children with BPD progressively increases with age [16]. Increased thickness, tortuosity and an irregular distribution of elastic fibers have been observed in children with BPD [23]. Increased elastic fiber content, uneven lung inflation, inflammation and edema have also been observed in premature lambs following prolonged mechanical ventilation for 3 to 4 weeks [13].

Regarding the deposition of collagen fibers, a quantitative increase and maturation of collagen fibers in the parenchyma occurs during normal lung development. The concentration and organization of collagen content is altered in experimental models of BPD. The proportion of collagen fibers relative to the lung parenchyma in response to oxygen exposure remains controversial in the literature. An increased deposition of collagen fibers was reported in children who are at risk for developing fatal cases of BPD [16]. A larger deposition of type I collagen fibers was found in the alveolar walls [48].

In the present study, the proportion of collagen fibers was increased in animals exposed to oxygen compared with those exposed to room air.

The major limitation of this study is that it was an experimental laboratory investigation and does not precisely reproduce the pathophysiology of BPD as it occurs in clinical practice. The lesions found in premature newborns usually occur due to mechanical ventilation with FiO_2_ values from 35% to 45%. This study reproduced BPD by maximizing the effects of oxygen toxicity, using a substantially higher level of FiO_2_ (80% and 95%), which the newborn infant does not experience. Another limitation of this study is that it did not investigate gestational age and oxygen concentration separately but determined the association of these two variables.

Understanding the pathophysiological mechanisms of BPD is vital for developing effective treatments for this disease. Hence, it is important to develop experimental models that enable researchers to better reproduce the pathology, to understand BPD more completely and to test new treatment methods.

This study has described an animal model that was developed to study BPD based on lung immaturity (29-day gestation) and oxygen exposure (80%), which can facilitate new studies involving oxygen-induced tissue injury. We have reproduced anatomopathological lesions comparable to those found in BPD in a simple and inexpensive manner, using only hyperoxia. We stress that this experimental animal model of oxygen lung injury is not superior to those that already exist, but it has technical features (including a lower cost and size of preterm animals [33±5 g]) that make it an additional option for studying BPD.

In conclusion, lung injury induced by prolonged oxygen exposure in premature rabbits born at 29 days of gestation and exposed to FiO_2_  = 80% resulted in a reduced number of alveoli and a trend in other histopathological findings comparable with those of BPD in humans. This group of animals had a higher survival rate compared with the group of animals born at 28 days of gestation.
